# Supplementary material for: Boolean modeling of mechanosensitive epithelial to mesenchymal transition and its reversal
Source: iScience. 2023 Mar 2;26(4):106321. doi: 10.1016/j.isci.2023.106321 (PMC10030917; doi:10.1016/j.isci.2023.106321)
Supplement: Document S1. Figures S1–S20 [file mmc1.pdf]

iScience, Volume *26*

Supplemental information

Boolean modeling of mechanosensitive epithelial to mesenchymal transition and its reversal

Emmalee Sullivan, Marlayna Harris, Arnav Bhatnagar, Eric Guberman, Ian Zonfa, and Erzsébet Ravasz Regan

Supplementary Figures

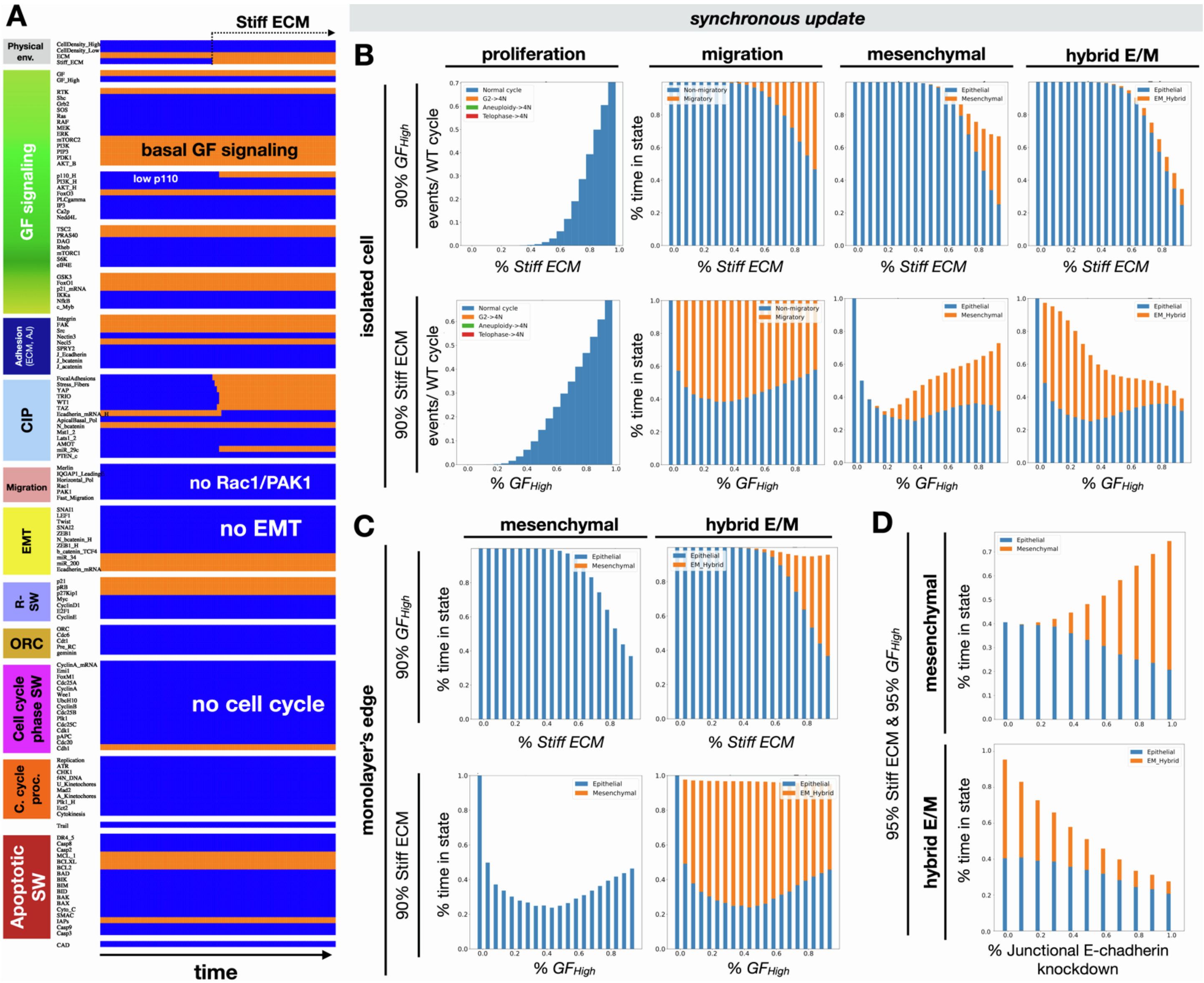

Figure S1. Stiff ECM, strong growth signals, and loss of adherens junctions are all required for mechanically induced EMT; related to Figure 2. A) Synchronous dynamics of regulatory molecule expression/activity during exposure of isolated, epithelial cells in low growth conditions to stiff ECM. *X-axis*: time-steps; *y-axis:* nodes organized in regulatory modules; *orange/blue:* ON/OFF; *black/white labels*: relevant molecular patterns. **B)** *Top row:* response of isolated cells to increasing *Stiff ECM* exposure in the presence of 90% saturating growth stimuli. *Top leftmost graphs:* Rate of normal cell cycle completion (*blue*), G2 → G1 reset followed by genome duplication (*orange*, not observed), aberrant mitosis followed by genome duplication (*green*, not observed), and failed cytokinesis followed by genome duplication (*red*, not observed) relative to the minimum cell cycle length (21 time-steps), shown as stacked bar charts. *Top row, 3 rightmost graphs:* fraction of time spent in a *i)* migratory (*orange*) vs. non-migratory (*blue*) state, *ii)* mesenchymal (*orange*) vs. epithelial (*blue*) state, and iii) hybrid E/M (*orange*) vs. epithelial (*blue*) state. *Bottom row:* parallel measurements as a function of increasing growth factor exposure on 90% stiff ECM. **C)** *Top/bottom:* response of cells at a monolayer's edge to increasing *Stiff ECM* exposure in the presence of 90% saturating growth stimuli (*top*) or increasing growth factor exposure on 90% stiff ECM (*bottom*). *Left/right:* fraction of time spent in a mesenchymal (*orange*) vs. epithelial (*blue*) state (*left*), or hybrid E/M (*orange*) vs. epithelial (*blue*) state (*right*). **D)** *Top/bottom:* fraction of time spent in a mesenchymal (*orange*) vs. epithelial (*blue*) state (*top*), or hybrid E/M (*orange*) vs. epithelial (*blue*) state (*bottom*) as a function of increasing *junctional E-cadherin* inhibition at a monolayer's edge, 95% *Stiff ECM* and 95% saturating growth stimuli. *Total sampled live cell time:* 100,000 steps; synchronous update; *Initial state for sampling runs:* isolated epithelial cell in low mitogens on a soft ECM.

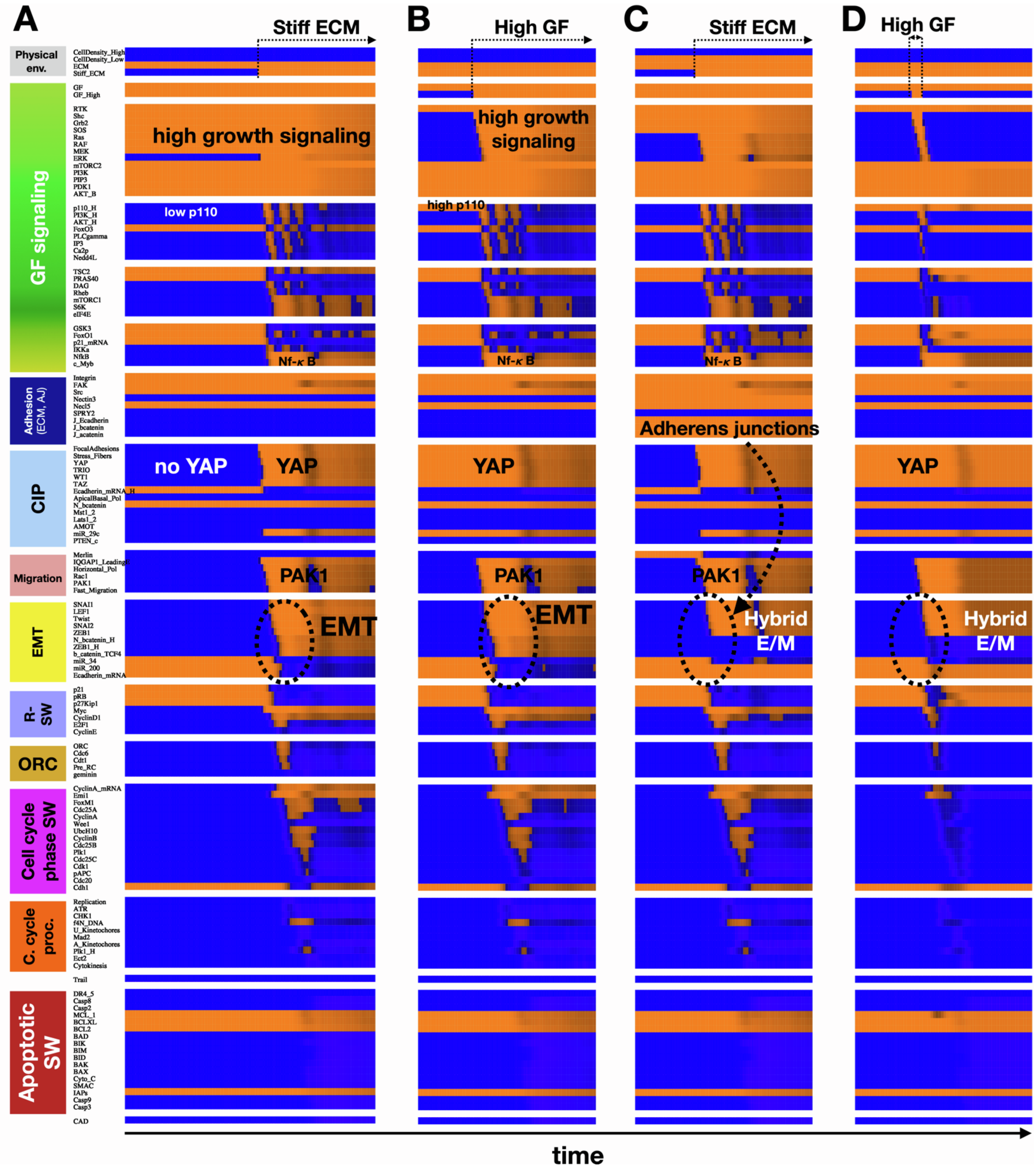

Figure S2. Partial vs. full EMT driven by growth signals on a stiff matrix at medium vs. low density is robust to biased asynchronous update; related to Figure 2. A-D) Biased asynchronous dynamics of regulatory molecule expression/activity during exposure of (A) isolated, growth-stimulated epithelial cells to stiff ECM, (B) isolated epithelial cells in low growth conditions on a stiff ECM to strong mitogens, (C) isolated, growth-stimulated epithelial cells at a monolayer edge to stiff ECM, and (D) isolated epithelial cells in low growth conditions on a stiff ECM to a brief (6-step) pulse of strong mitogens. *X-axis:* time-steps; *y-axis:* nodes organized in regulatory modules; *orange/black/blue color-scale:* average expression of each molecule across 1000 independent runs with biased asynchronous update (*orange* = all ON; *black* = 50% ON/OFF; *blue* = all OFF); *black/white labels & arrows:* molecular changes that drive EMT.

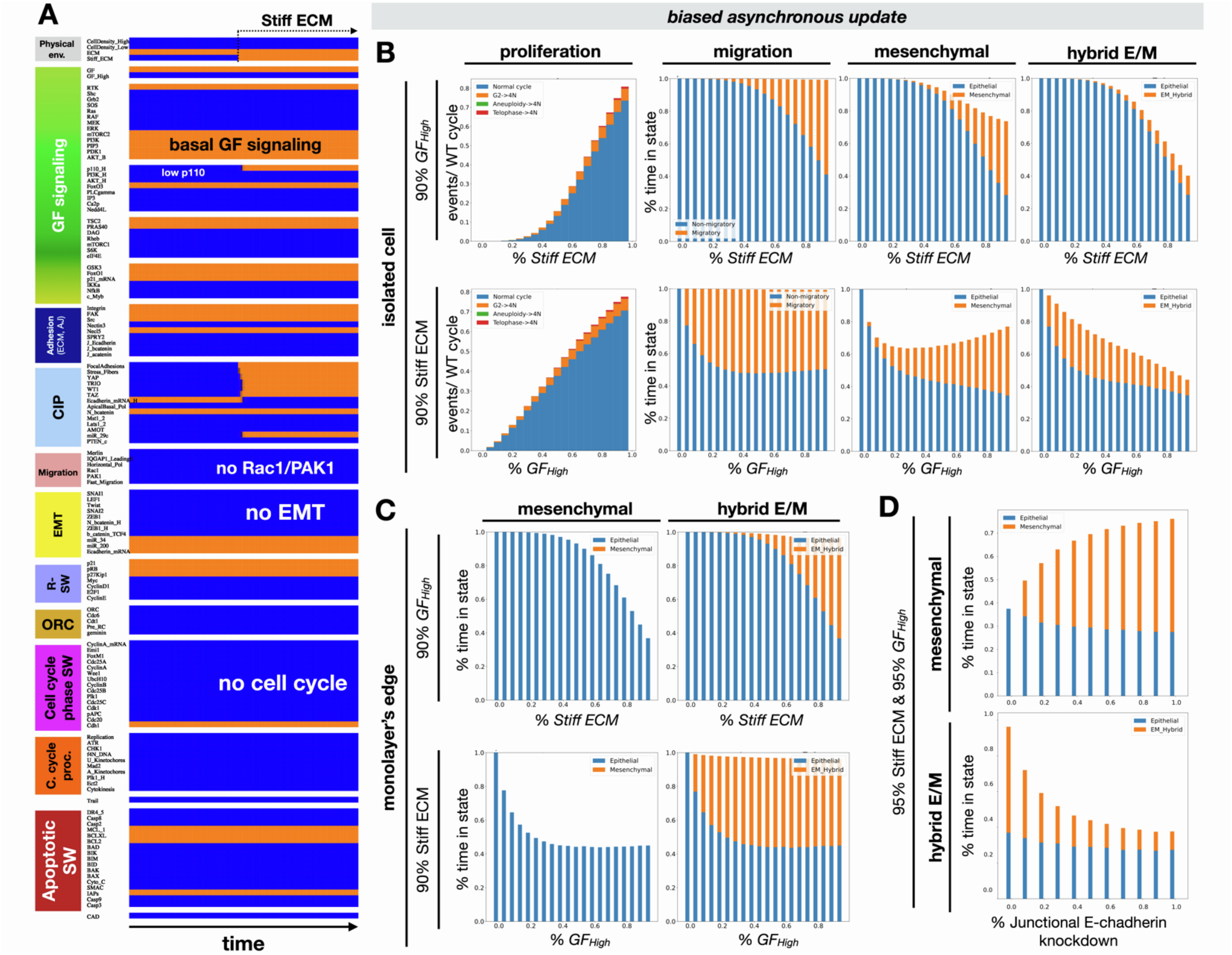

Figure S3. Mechanically induced EMT requiring stiff ECM, growth signals, and loss of adherens junctions is robust to biased asynchronous update; related to Figure 2. A) Biased asynchronous dynamics of regulatory molecule expression/activity during exposure of isolated, epithelial cells in low growth conditions to stiff ECM. *X-axis*: time-steps; *y-axis:* nodes organized in regulatory modules; *orange/black/blue color-scale:* average expression of each molecule across 1000 independent runs with biased asynchronous update (*orange* = all ON; *black* = 50% ON/OFF; *blue* = all OFF); *black/white labels*: relevant molecular patterns. **B)** *Top row:* response of isolated cells to increasing *Stiff ECM* exposure in the presence of 90% saturating growth stimuli. *Top leftmost graphs:* Rate of normal cell cycle completion (*blue*), G2 → G1 reset followed by genome duplication (*orange*), aberrant mitosis followed by genome duplication (*green*, not observed), and failed cytokinesis followed by genome duplication (*red*) relative to the minimum synchronous cell cycle length (21 time-steps), shown as stacked bar charts. *Top row, 3 rightmost graphs:* fraction of time spent in a *i)* migratory (*orange*) vs. non-migratory (*blue*) state, *ii)* mesenchymal (*orange*) vs. epithelial (*blue*) state, and iii) hybrid E/M (*orange*) vs. epithelial (*blue*) state. *Bottom row:* parallel measurements as a function of increasing growth factor exposure on 90% stiff ECM. **C)** *Top/bottom:* response of cells at a monolayer's edge to increasing *Stiff ECM* exposure in the presence of 90% saturating growth stimuli (*top*) or increasing growth factor exposure on 90% stiff ECM (*bottom*). *Left/right:* fraction of time spent in a mesenchymal (*orange*) vs. epithelial (*blue*) state (*left*), or hybrid E/M (*orange*) vs. epithelial (*blue*) state (*right*). **D)** *Top/bottom:* fraction of time spent in a mesenchymal (*orange*) vs. epithelial (*blue*) state (*top*), or hybrid E/M (*orange*) vs. epithelial (*blue*) state (*bottom*) as a function of increasing *junctional E-cadherin* inhibition at a monolayer's edge, 95% *Stiff ECM* and 95% saturating growth stimuli. *Total sampled live cell time:* 100,000 steps; synchronous update; *Initial state for sampling runs:* isolated epithelial cell in low mitogens on a soft ECM.

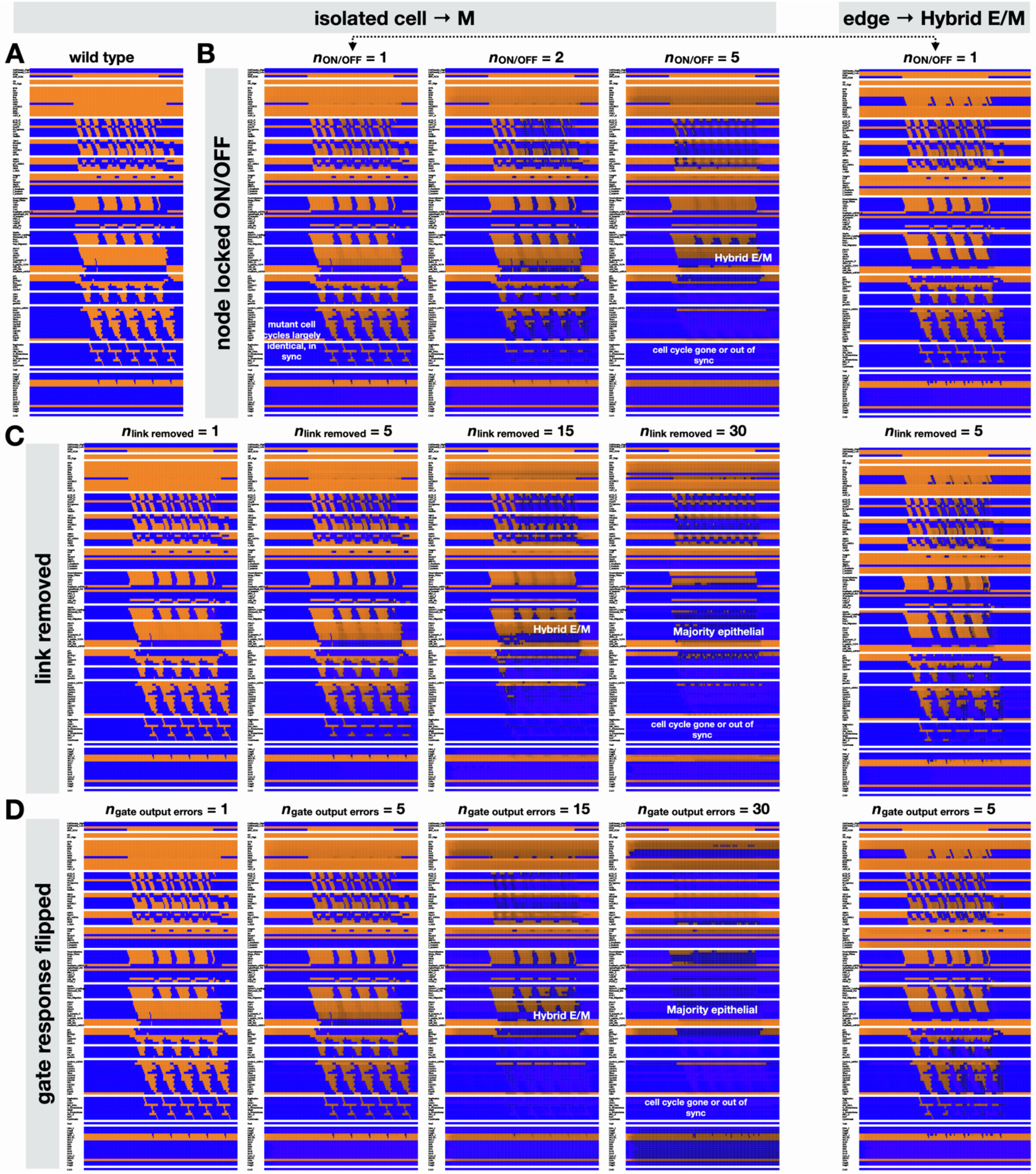

Figure S4. Mechanosensitive EMT and MET are robust to random mutations / errors in model construction; related to Figure 2. A) Synchronous dynamics of regulatory molecule expression/activity during exposure of an isolated, growth-stimulated epithelial cell to stiff ECM (wild-type model). **B-D)** *Leftmost panels:* simulation in (A) averaged over 1000 distinct mutant networks with (B) $n \in \{1,2,5\}$ random nodes per network locked ON or OFF, (C) $n \in \{1,5,15,30\}$ random links per network removed, and (D) $n \in \{1,5,15,30\}$ random gate outputs per network flipped. *Right column:* matching experiments starting with a cell at a monolayer's edge with (B) one locked node, (C) 5 links removed, or (D) 5 gate outputs flipped. *X-axis*: time-steps; *y-axis:* nodes organized in regulatory modules; *orange/black/blue color-scale:* average expression of each molecule across 1000 time-courses from independently generated mutant models (*orange* = all ON; *black* = 50% ON/OFF; *blue* = all OFF; synchronous update); *black/white labels*: relevant molecular patterns.

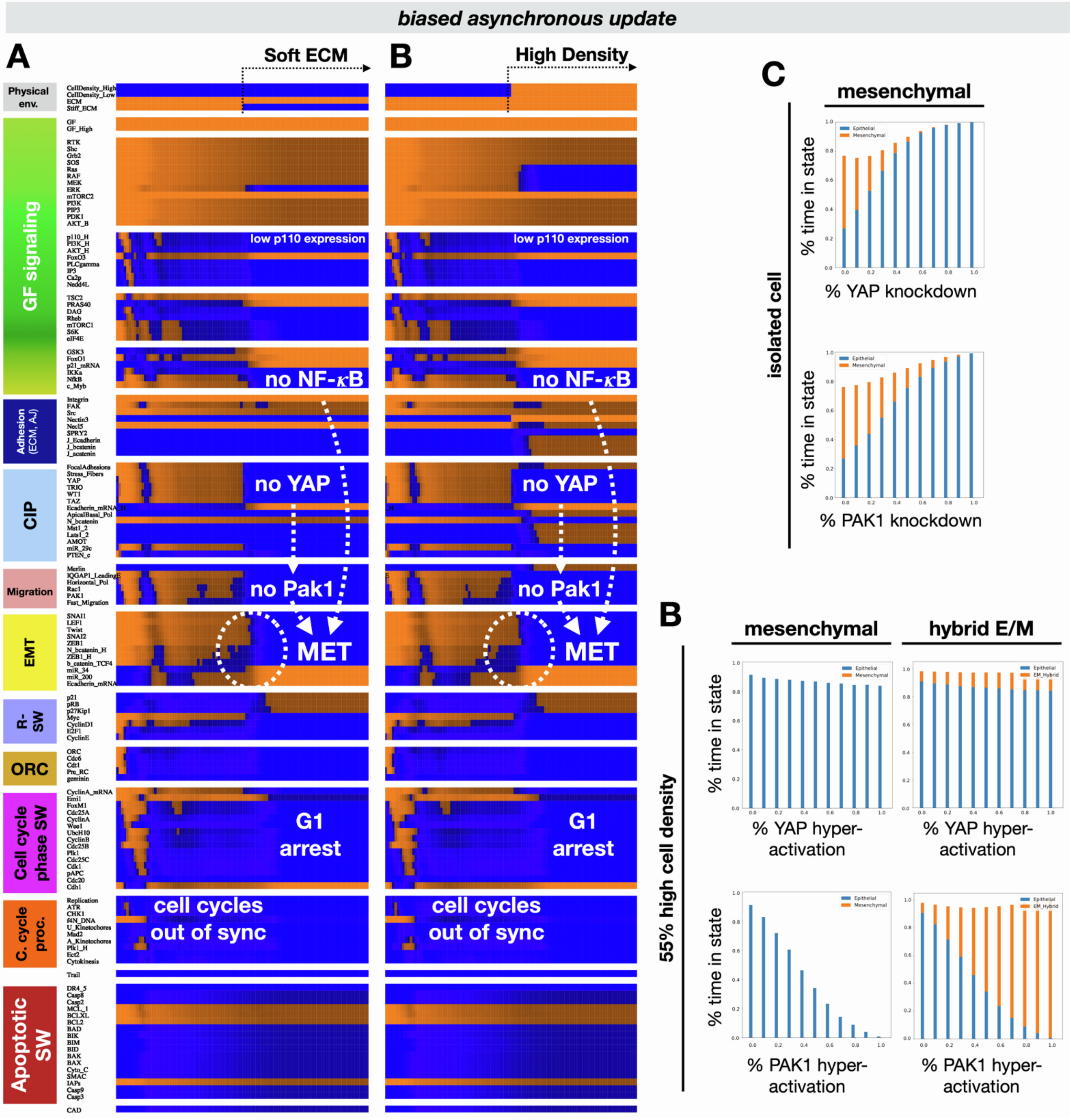

Figure S5. MET triggered by soft matrix exposure and high cell density in the absence of autocrine or external EMT-promoting signals is robust to biased asynchronous update; related to Figure 3. A-B) Biased asynchronous dynamics of regulatory molecule expression/activity during exposure of an isolated, growth-stimulated and proliferating mesenchymal cell to (A) soft ECM, and (B) very high cell density. *X-axis:* time-steps; *y-axis:* nodes organized in regulatory modules; *orange/black/blue color-scale:* average expression of each molecule across 1000 independent runs with biased asynchronous update (*orange* = all ON; *black* = 50% ON/OFF; *blue* = all OFF); *black/white labels & arrows:* molecular changes that drive MET. **C)** *Top/bottom:* fraction of time spent in a mesenchymal (*orange*) vs. epithelial (*blue*) state as a function of increasing *YAP1* (*top*) or *PAK1* (*bottom*) inhibition in isolated cells on 95% *Stiff ECM* and exposed to 95% saturating growth stimuli. **D)** *Top/bottom:* fraction of time spent in *i*) mesenchymal (*orange*) vs. epithelial (*blue*) state (*left*) or *ii*) hybrid E/M (*orange*) vs. epithelial (*blue*) state (*right*) as a function of increasing *YAP1* (*top*) or *PAK1* (*bottom*) inhibition in cells at 55% high density (no free space to spread 55% of the time) on 95% *Stiff ECM* and exposed to 95% saturating growth stimuli. *Total sampled live cell time:* 100,000 steps; synchronous update; *Initial state for sampling runs:* isolated epithelial cell in low mitogens on a soft ECM.

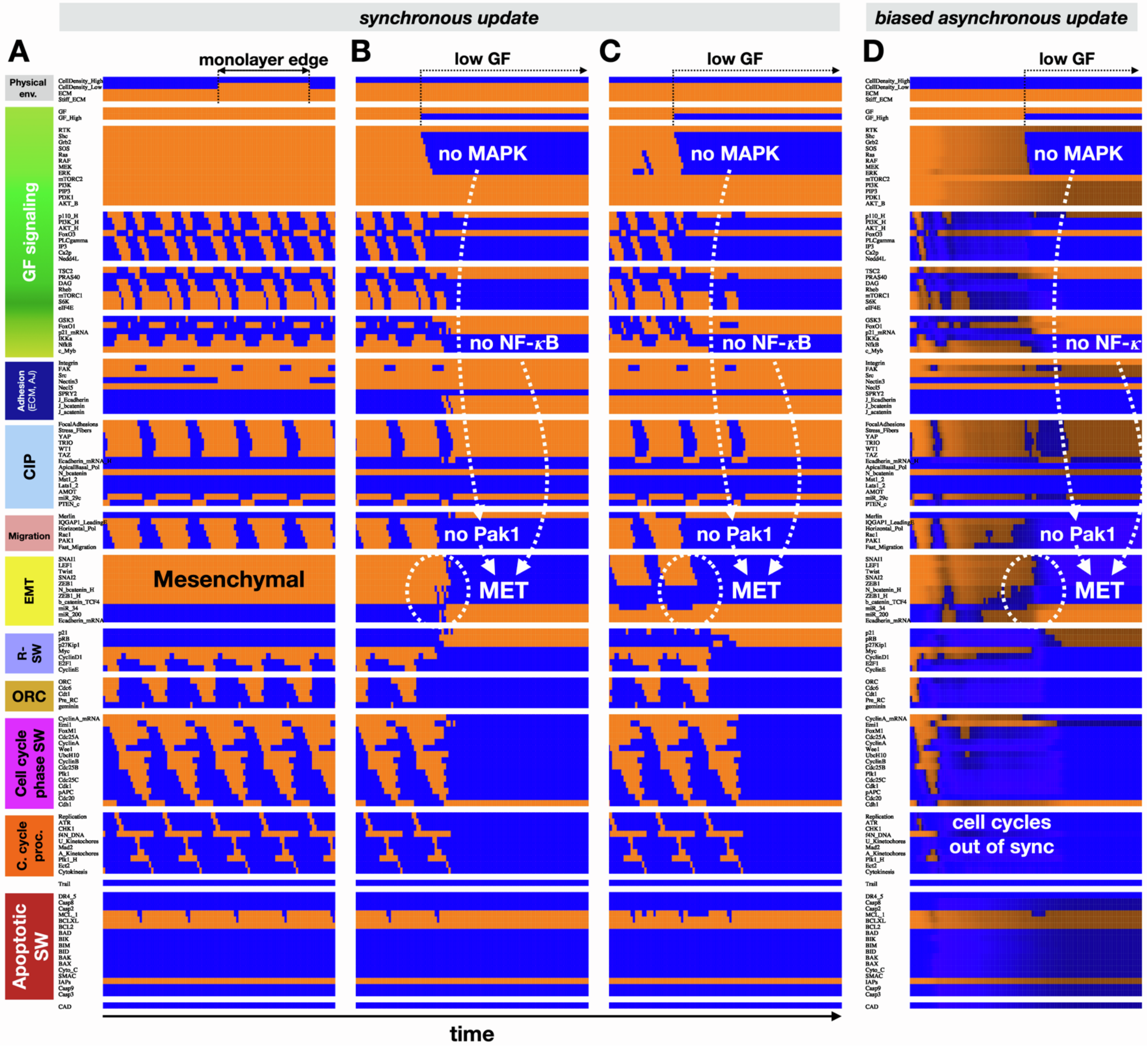

Figure S6. Mitogen withdrawal triggers MET in proliferating mesenchymal and hybrid E/M cells without autocrine *TGFβ* signaling; related to Figure 3. A-C) Synchronous dynamics of regulatory molecule expression/activity during exposure of (A) isolated, growth-stimulated mesenchymal cells to densities akin to a monolayer's edge, (B) dividing mesenchymal cells at a monolayer's edge to a decrease in growth signals, and (C) dividing hybrid E/M cells at a monolayer's edge to a decrease in growth signals. **D)** Biased asynchronous dynamics of regulatory molecule expression/activity during exposure of dividing mesenchymal cells at a monolayer's edge to a decrease in growth signals. *X-axis:* time-steps; *y-axis:* nodes organized in regulatory modules; *orange/black/blue color-scale:* average expression of each molecule across 1000 independent runs with biased asynchronous update (*orange* = all ON; *black* = 50% ON/OFF; *blue* = all OFF); *black/white labels & arrows:* molecular changes that drive MET.

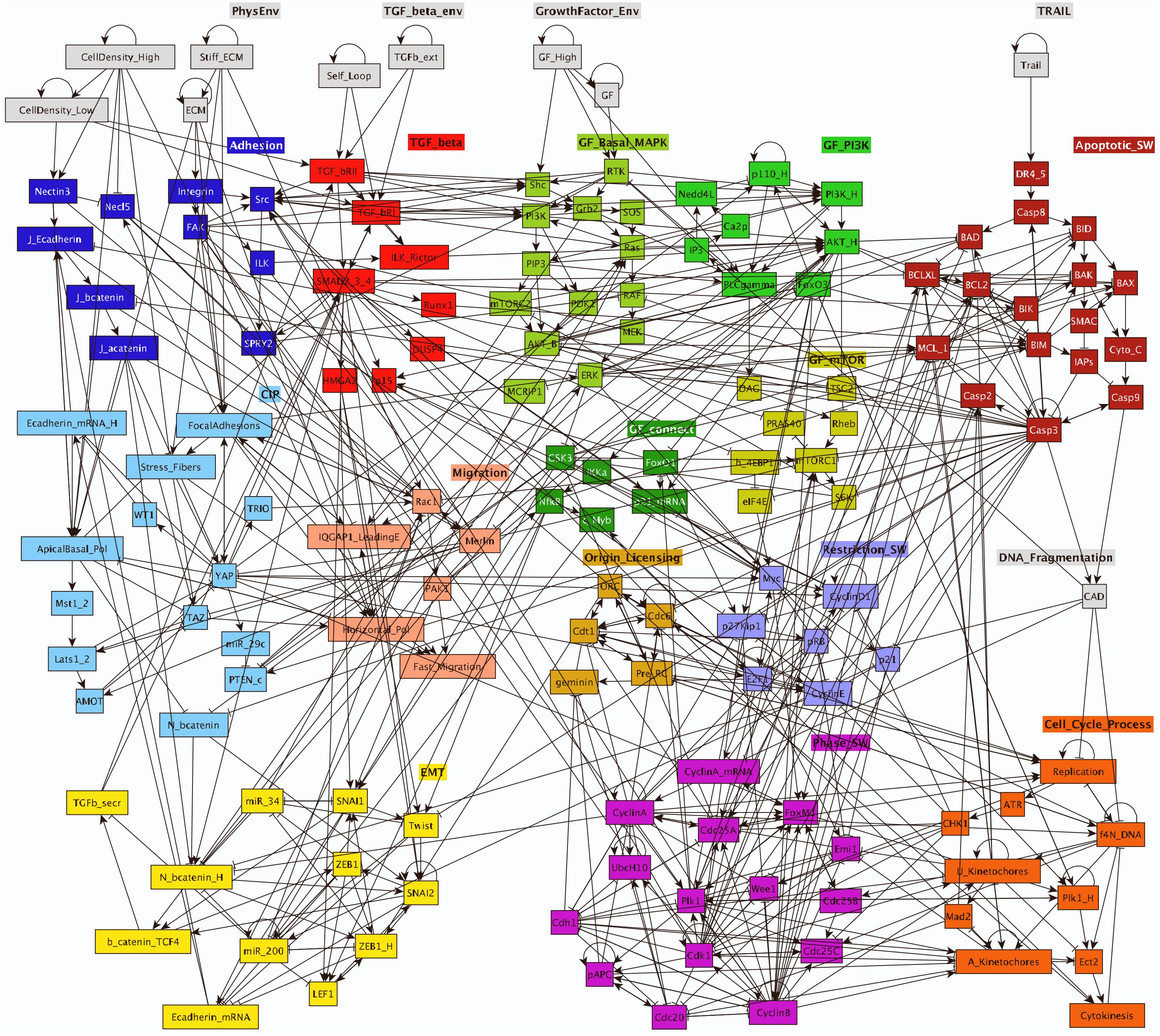

Figure S7. Modular model of *TGFβ* signaling, EMT, growth factor signaling, cell cycle and apoptosis, adhesion, adherens junction formation, contact inhibition, and migration; related to Figure 4. Modular network representation of our extended Boolean model. *Gray:* inputs representing environmental factors; *dark blue:* Adhesion signals; *red: TGFβ* signaling; *green*: Growth Signaling (*lime green*: basal AKT & MAPK, *bright green:* PI3K/AKT oscillations, *mustard*: mTORC1, *dark green: NF-κB, GSK3, FoxO1*); *dark red:* Apoptotic Switch*; light blue:* Contact Inhibition; *pink/light orange:* Migration; *light brown:* Origin of Replication Licensing; *lilac:* Restriction Switch; *purple:* Phase Switch; *dark orange:* cell cycle processes; *yellow:* EMT switch; → : activation; ⊣ : inhibition.

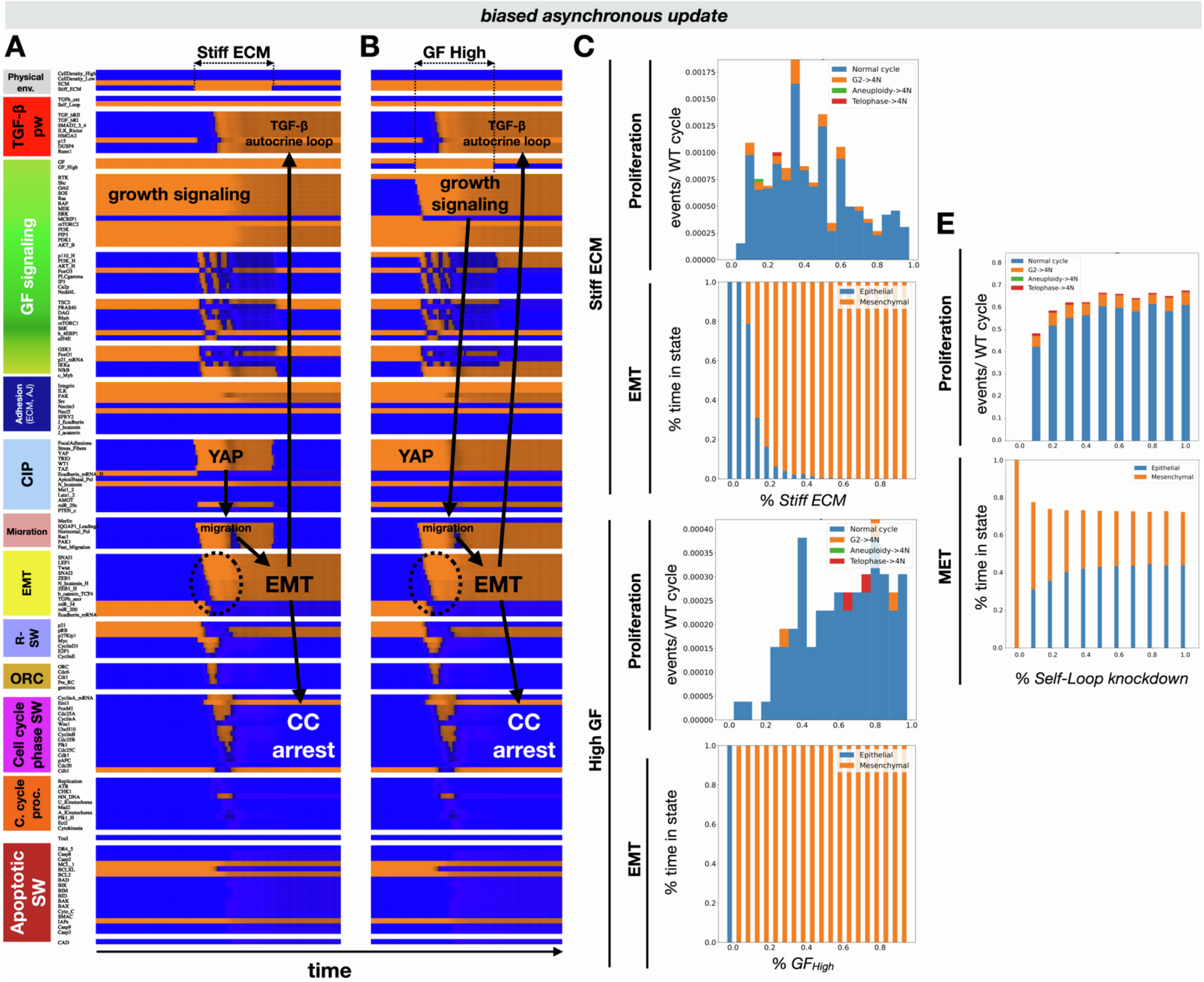

Figure S8. Autocrine TGFβ-mediated stable commitment to a mesenchymal state at very low density with stiff ECM and growth signaling is robust biased asynchronous update; related to Figure 4. A, B) Biased asynchronous dynamics of regulatory molecule expression/activity during exposure of (B) an isolated, growth-stimulated epithelial cell to stiff ECM, and (C) an isolated epithelial cell on stiff ECM to strong mitogens. *X-axis:* time-steps; *y-axis:* nodes organized in regulatory modules; *orange/black/blue color-scale:* average expression of each molecule across 1000 independent runs with biased asynchronous update (*orange* = all ON; *black* = 50% ON/OFF; *blue* = all OFF); *black/white labels & arrows:* molecular changes that drive EMT. **C)** *Top two vs. bottom 2 panels:* response of isolated cells to *i*) increasing *Stiff ECM* exposure in the presence of 95% saturating growth stimuli (*top two*) vs. ii) increasing growth factor exposure on 95% stiff ECM (*bottom two*). *Proliferation label:* rate of normal cell cycle completion (*blue*) vs. G2 → G1 reset (*orange*), aberrant mitosis (*green*, not observed), or failed cytokinesis (*red*) followed by genome duplication, relative to the minimum cell cycle length (21 time-steps), shown as stacked bar charts. *EMT label:* fraction of time spent in a mesenchymal (*orange*) vs. epithelial (*blue*) state. **D)** Response of isolated cells on 85% Stiff ECM exposed to 85% saturating growth stimuli to increasing inhibition of autocrine signaling (*Self-Loop* knockdown). *Top:* rate of normal cell cycle completion (*blue*) vs. G2 → G1 reset (*orange*), aberrant mitosis (*green*, not observed), or failed cytokinesis (*red*) followed by genome duplication. *Bottom:* fraction of time spent in a mesenchymal (*orange*) vs. epithelial (*blue*) state. *Total sampled live cell time:* 100,000 steps; synchronous update; *Initial state for sampling runs:* isolated epithelial cell in low mitogens on a soft ECM.

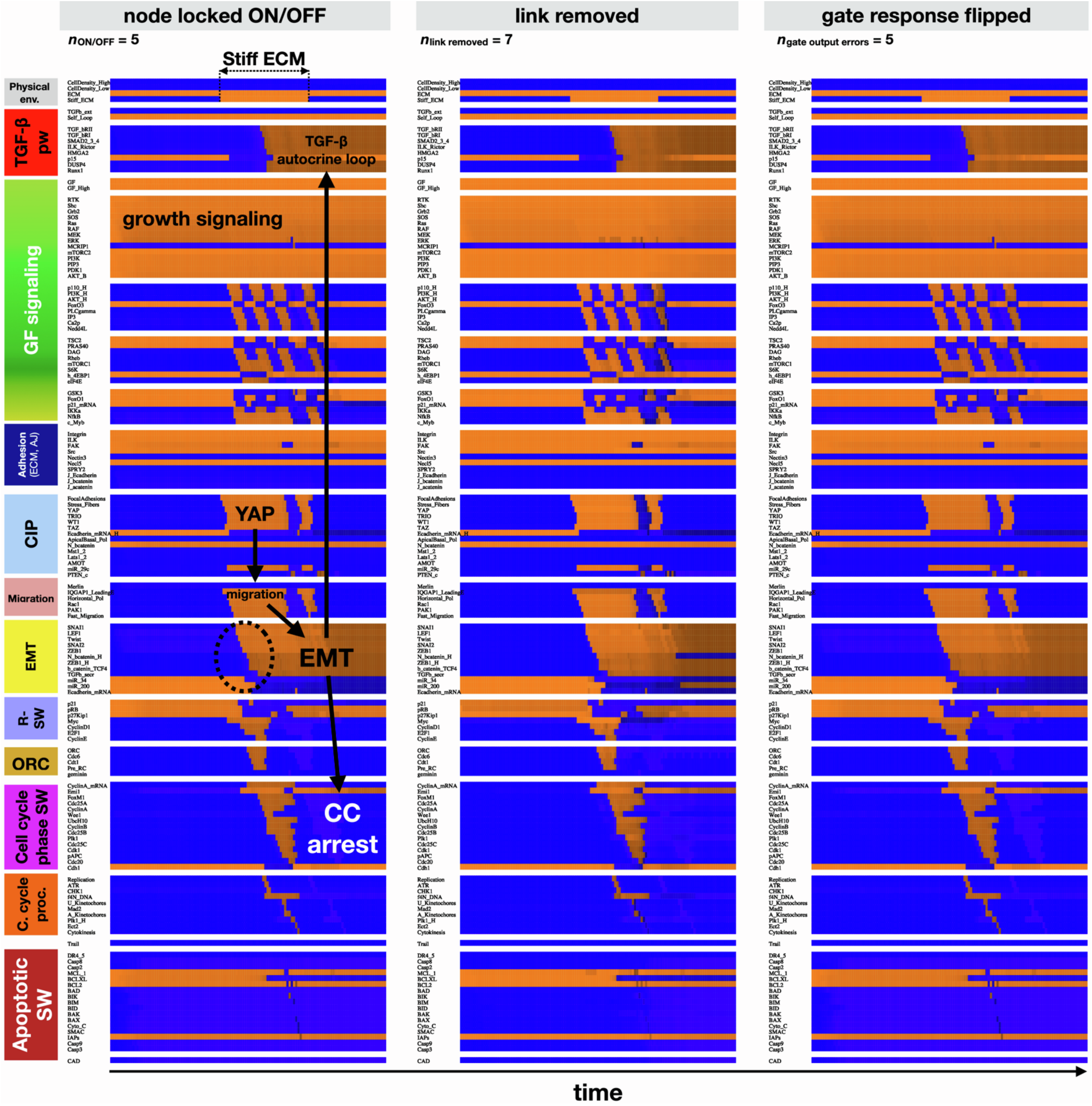

Figure S9. Stabilization of EMT in response to stiff ECM by autocrine *TGFβ* is robust to random mutations / errors in model construction; related to Figure 4. Average synchronous dynamics of regulatory molecule expression/activity during reversible exposure of an isolated, growth-stimulated epithelial cell to stiff ECM in an ensemble of mutant networks with 5 random nodes locked ON/OFF (*left*), 7 random links removed (*middle*), and 5 random gate outputs flipped per network (*right*). *X-axis*: time-steps; *y-axis:* nodes organized in regulatory modules; *orange/black/blue color-scale:* average expression of each molecule across 1000 time-courses from independently generated mutant models (*orange* = all ON; *black* = 50% ON/OFF; *blue* = all OFF; synchronous update); *black/white labels*: relevant molecular patterns.

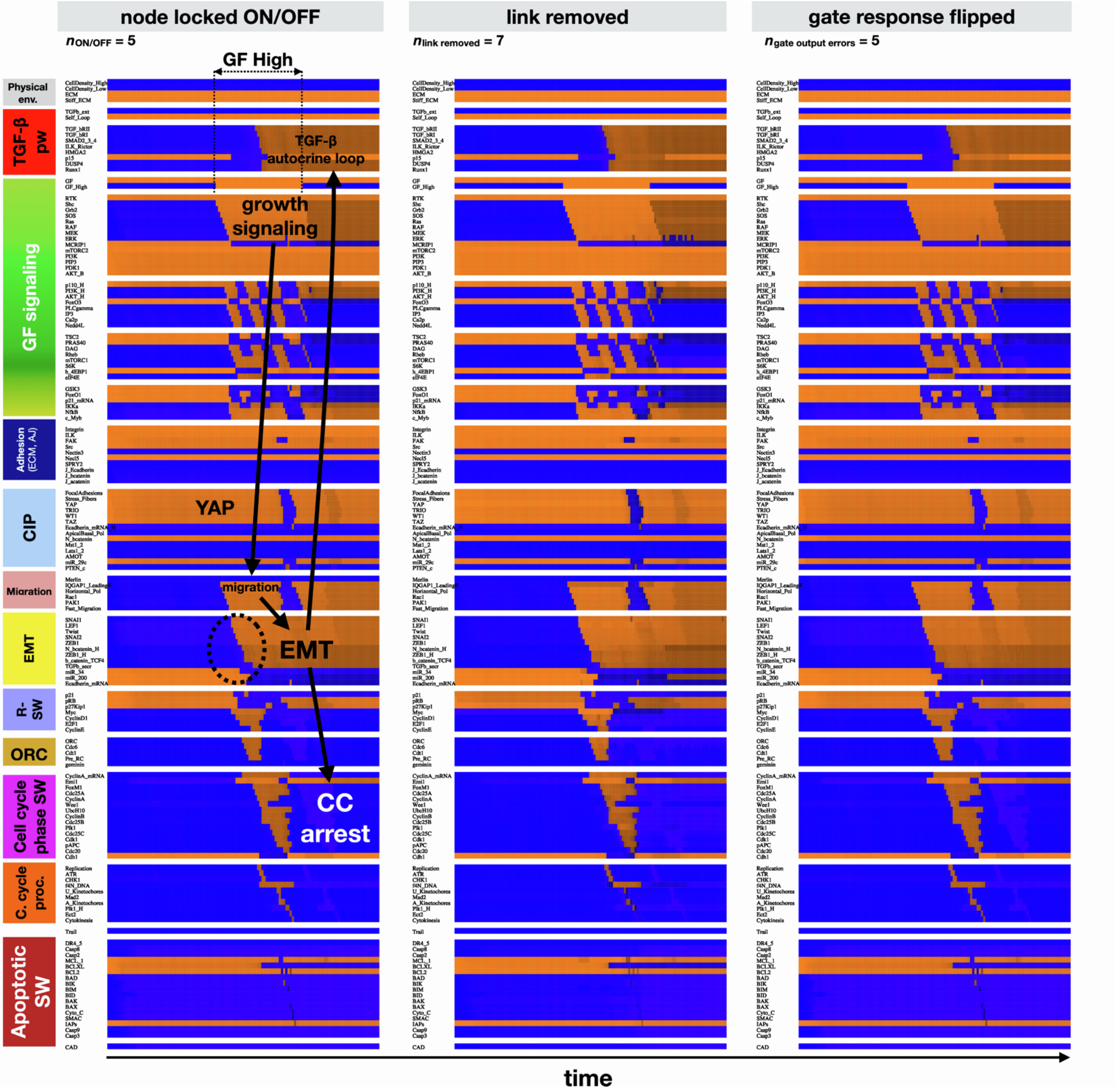

Figure S10. Stabilization of EMT in response to growth factors by autocrine *TGFβ* is robust to random mutations / errors in model construction; related to Figure 4. Average synchronous dynamics of regulatory molecule expression/activity during exposure of an isolated epithelial cell on a stiff ECM reversibly exposed to high levels of growth stimulus in an ensemble of mutant networks with 5 random nodes locked ON/OFF (*left*), 7 random links removed (*middle*), and 5 random gate outputs flipped per network (*right*). *X-axis*: time-steps; *y-axis:* nodes organized in regulatory modules; *orange/black/blue color-scale:* average expression of each molecule across 1000 time-courses from independently generated mutant models (*orange* = all ON; *black* = 50% ON/OFF; *blue* = all OFF; synchronous update); *black/white labels*: relevant molecular patterns.

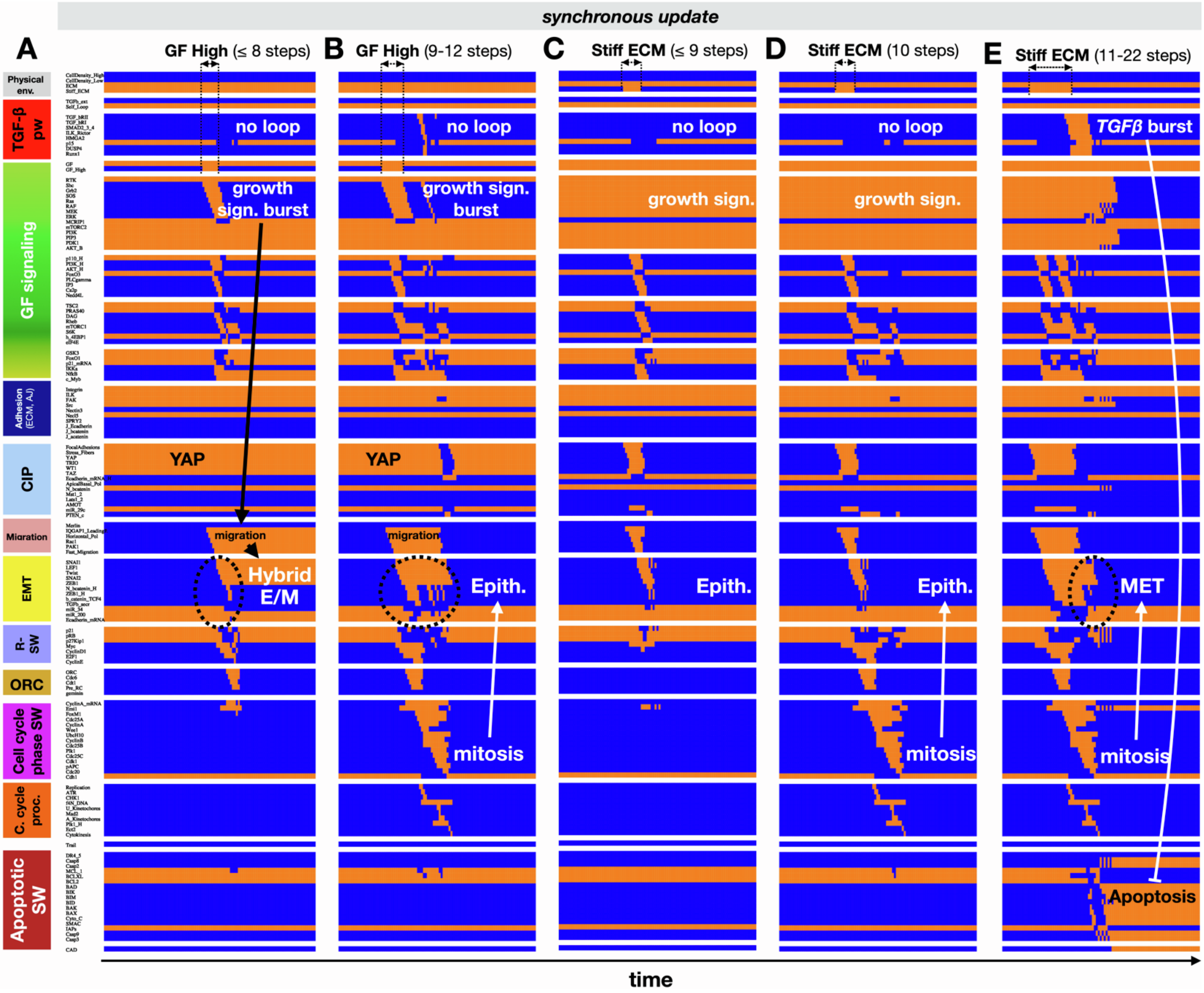

Figure S11. In isolated cells with strong autocrine *TGFβ* signaling, short-lived growth signal bursts can trigger sustained hybrid E/M or cell cycle and no EMT, while exposure to stiff ECM that stops before cell cycle arrest can trigger apoptosis; related to Figure 4. A-B) Synchronous dynamics of regulatory molecule expression/activity during exposure of isolated epithelial cells on stiff ECM to strong but brief growth signal bursts; (A): 8 timesteps leading to hybrid E/M; (B): 11 timesteps leading to a single division and reset to an epithelial state. **C-E)** Dynamics during exposure of isolated, growth-stimulated epithelial cells on soft ECM to stiff ECM for brief intervals; (C): 9 timesteps trigger no response; (D): 10 timesteps leading to a single division without EMT; (E): 22 timesteps leading to apoptosis. *X-axis:* time-steps; *y-axis:* nodes organized in regulatory modules; *orange/blue:* ON/OFF; *black/white labels & arrows:* molecular changes that drive MET.

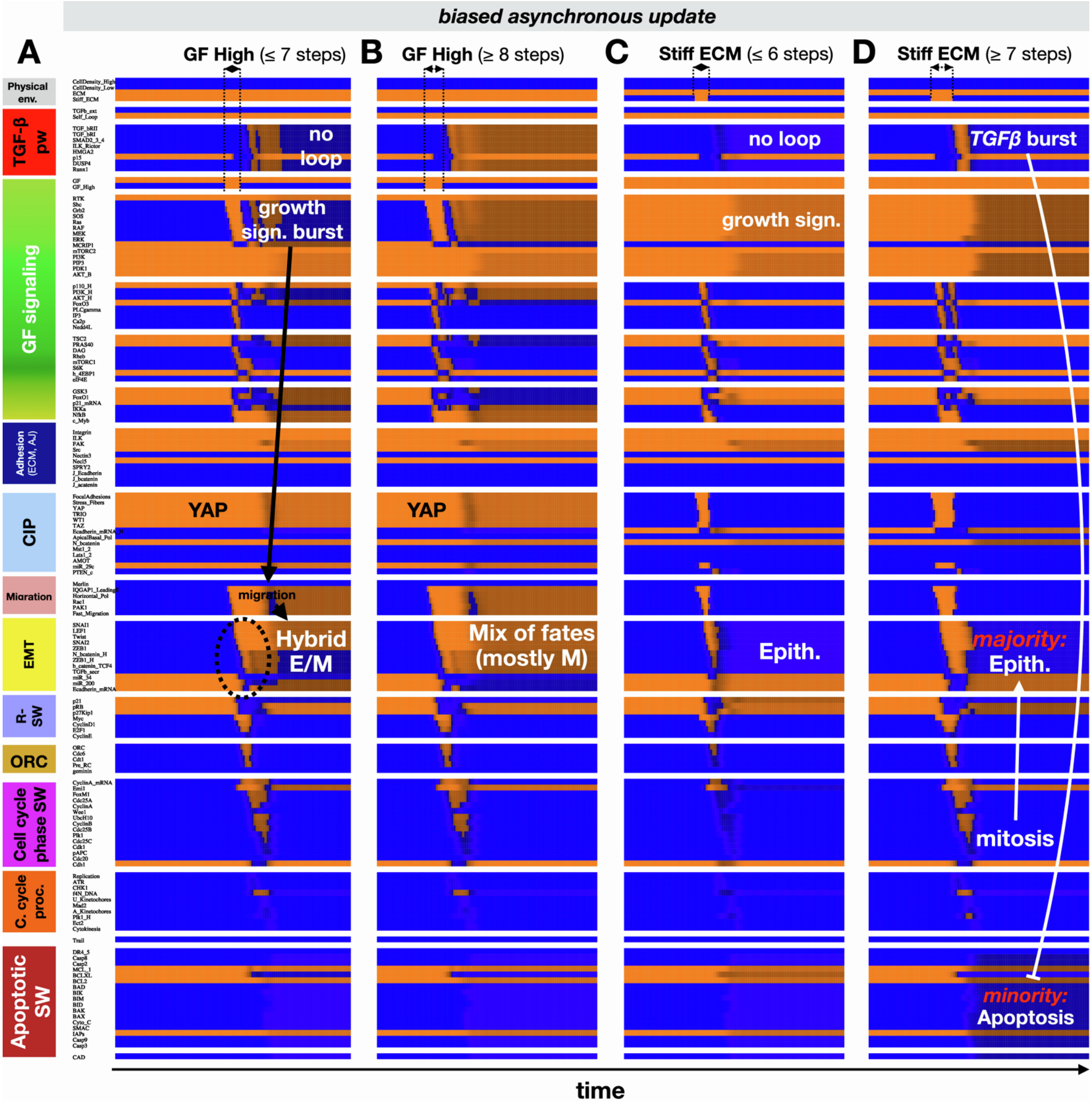

Figure S12. Simulations reproducing Figure S11 with biased asynchronous update (short-lived growth signals or stiff ECM exposure); related to Figure 4. A-B) Biased asynchronous dynamics of regulatory molecule expression/activity during exposure of isolated epithelial cells on stiff ECM to strong but brief growth signal bursts; (A): 7 timesteps leading to hybrid E/M; (B): 8 or more timesteps leading to a single division and a mix of fates, including mesenchymal and epithelial ones (dark orange/blue shading, towards black). **C-D)** Biased asynchronous dynamics during exposure of isolated, growth-stimulated epithelial cells on soft ECM to stiff ECM for brief intervals; (C): 6 timesteps trigger no response; (D): 7 or more timesteps lead to mix of fates, including a single division without EMT and apoptosis. *X-axis:* time-steps; *y-axis:* nodes organized in regulatory modules; *orange/black/blue color-scale:* average expression of each molecule across 1000 independent runs with biased asynchronous update (*orange* = all ON; *black* = 50% ON/OFF; *blue* = all OFF); *black/white labels & arrows:* molecular changes that drive MET.

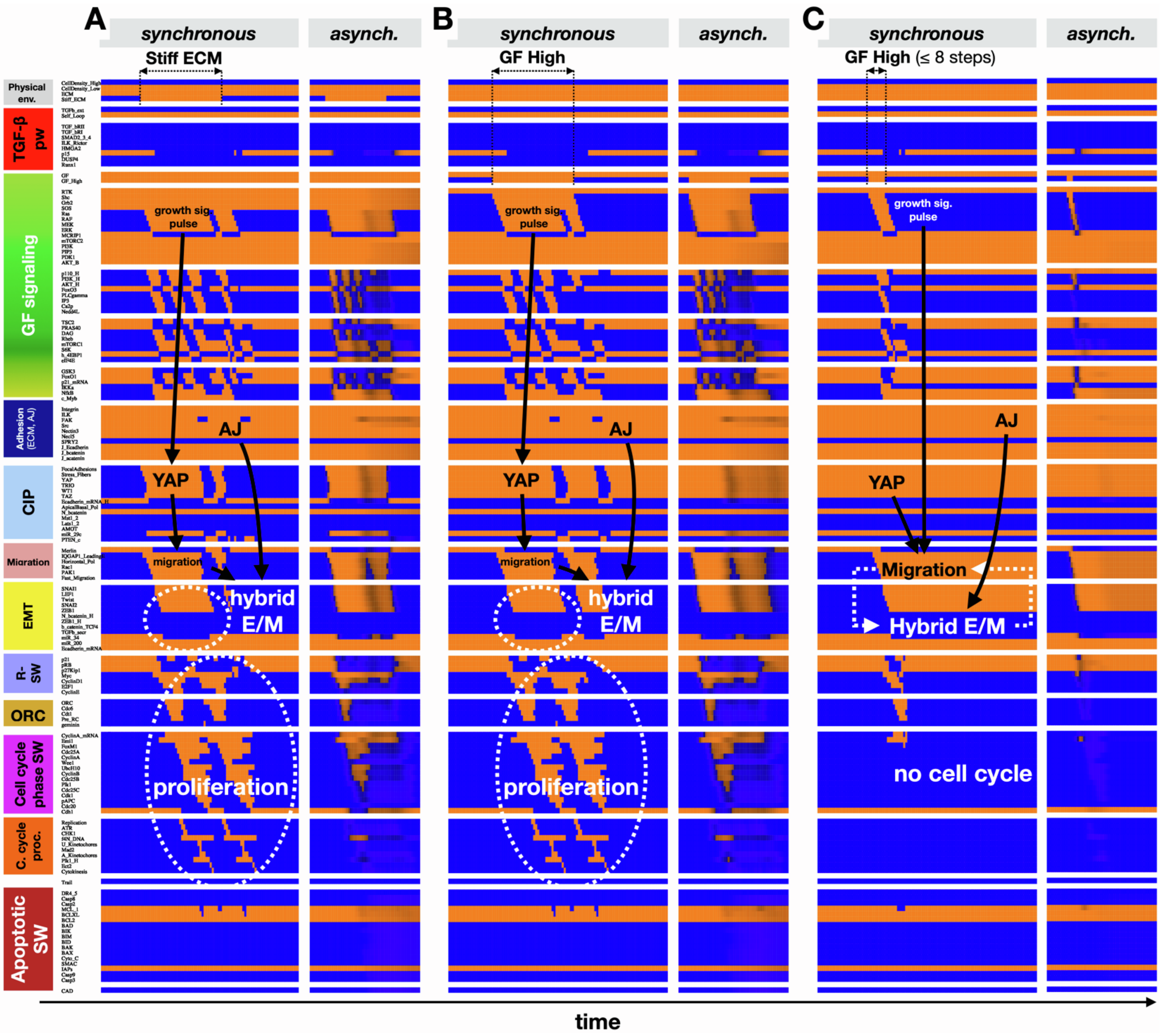

Figure S13. In cells at a monolayer's edge, exposure to stiff ECM and prolonged mitogens both trigger partial EMT to a proliferative, reversible hybrid E/M state, while short-lived growth signals can trigger sustained hybrid E/M; related to Figure 4. A) Synchronous (*left*) and biased asynchronous (*right*) dynamics of regulatory molecule expression/activity during exposure of growth-stimulated epithelial cells at a monolayer's edge on soft ECM to stiff ECM (40 timesteps), leading to reversible, proliferative hybrid E/M state. **B-C)** Synchronous (*left*) and biased asynchronous (*right*) dynamics during exposure of epithelial cells at a monolayer's edge on stiff ECM to strong growth signals; (B): 40 timesteps leading to reversible, proliferative hybrid E/M; (C): 8 synchronous (*left*) or 3 asynchronous (*right*) timesteps leading to a sustained hybrid E/M state. *X-axis:* time-steps; *y-axis:* nodes organized in regulatory modules; *orange/blue:* ON/OFF (*left*); *orange/black/blue color-scale:* average expression of each molecule across 1000 independent runs with biased asynchronous update (*orange* = all ON; *black* = 50% ON/OFF; *blue* = all OFF); *black/white labels & arrows:* molecular changes that drive EMT/MET.

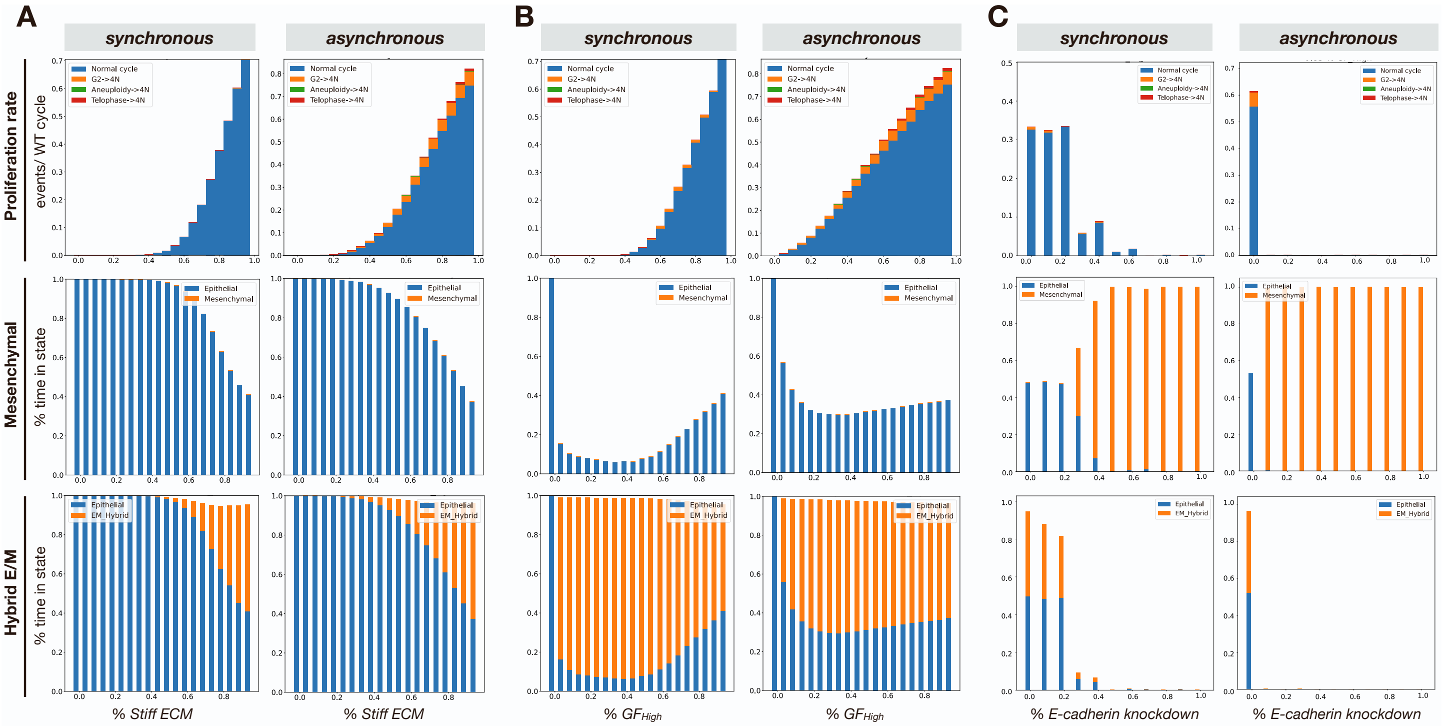

Figure S14. In cells at a monolayer's edge, stiff ECM and mitogens promote proliferation and the hybrid E/M state, while knockdown of E-cadherin pushes cells to a mesenchymal state; related to Figure 4. Synchronous (*left*) and biased asynchronous (*right*) response of cells at the edge of a monolayer cells to **A)** increasing *Stiff ECM* exposure in the presence of 95% saturating growth stimuli, **B)** increasing growth factor exposure on 95% stiff ECM, and **C)** increasing inhibition of *junctional E-cadherin* at 85% *Stiff ECM* and 85% saturating growth stimuli. *Left panels:* synchronous update; *right panels:* biased asynchronous update. *Top row (Proliferation):* rate of normal cell cycle completion (*blue*) vs. G2 → G1 reset (*orange*), aberrant mitosis (*green*), or failed cytokinesis (*red*) followed by genome duplication, relative to the minimum synchronous cell cycle length (21 time-steps), shown as stacked bar charts. *middle row (Mesenchymal):* fraction of time spent in a mesenchymal (*orange*) vs. epithelial (*blue*) state; *bottom row (Hybrid E/M):* fraction of time spent in a mesenchymal (*orange*) vs. epithelial (*blue*) state. *Total sampled live cell time:* 100,000 steps; synchronous update; *initial state for sampling runs:* isolated epithelial cell in low mitogens on a soft ECM.

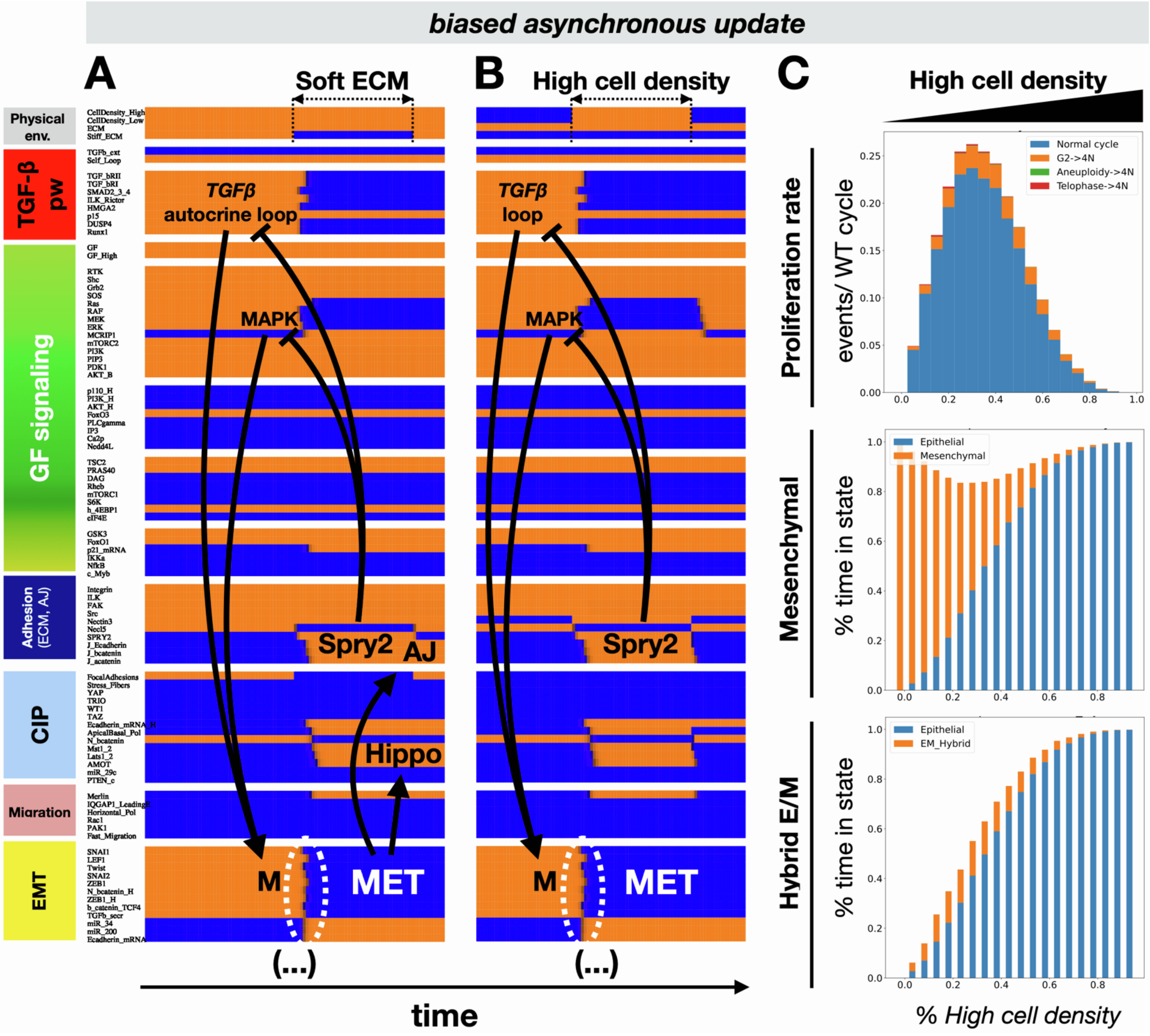

Figure S15. MET due to loss of autocrine TGFβ signaling on soft ECM at high cell density is robust to biased asynchronous update; related to Figure 5. A-B) Biased asynchronous dynamics of regulatory molecule expression/activity during exposure of (A) a growth-stimulated mesenchymal cell on a stiff matrix but at very high density to a soft ECM, and (B) an isolated mesenchymal cell on soft ECM to high density. *X-axis:* time-steps; *y-axis:* nodes organized in regulatory modules; *orange/black/blue color-scale:* average expression of each molecule across 1000 independent runs with biased asynchronous update (*orange* = all ON; *black* = 50% ON/OFF; *blue* = all OFF); *black/white labels & arrows:* molecular changes that drive MET. **C)** Response of mesenchymal cells at the edge of a monolayer to increasing cell density in the presence of 95% saturating growth stimuli on 95% stiff ECM. *Top:* rate of normal cell cycle completion (*blue*) vs. G2 → G1 reset (*orange*), aberrant mitosis (*green*), or failed cytokinesis (*red*) followed by genome duplication, relative to the minimum cell cycle length (21 time-steps), shown as stacked bar charts. *Middle:* fraction of time spent in a mesenchymal (*orange*) vs. epithelial (*blue*) state. *Bottom:* fraction of time spent in a hybrid E/M (*orange*) vs. epithelial (*blue*) state. *Total sampled live cell time:* 100,000 steps; synchronous update; *Initial state for sampling runs:* isolated epithelial cell in low mitogens on a soft ECM.

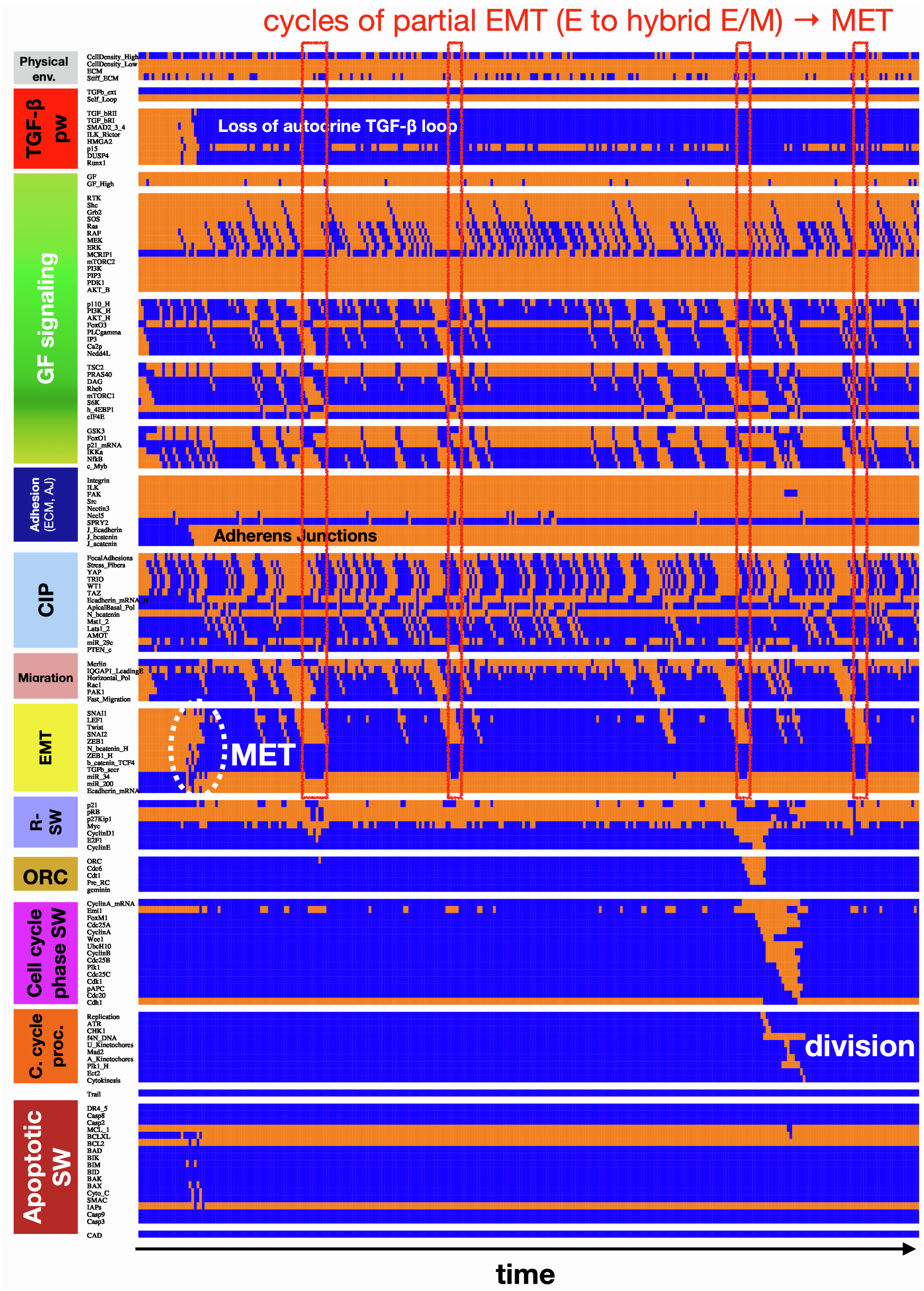

Figure S16. Transitioning from mesenchymal to an epithelial - hybrid E/M mix requires full MET followed by reversible partial EMT; related to Figure 5. Dynamics of regulatory molecule expression/activity of an initially mesenchymal cell on 75% stiff ECM at 20% high cell density (cell has no room to spread 20% of the time), showing irreversible EMT (white oval) followed by occasional partial EMT (E to hybrid E/M) and its reversal (red rectangles). *X-axis:* time-steps; *y-axis:* nodes organized in regulatory modules; *orange/blue:* ON/OFF.

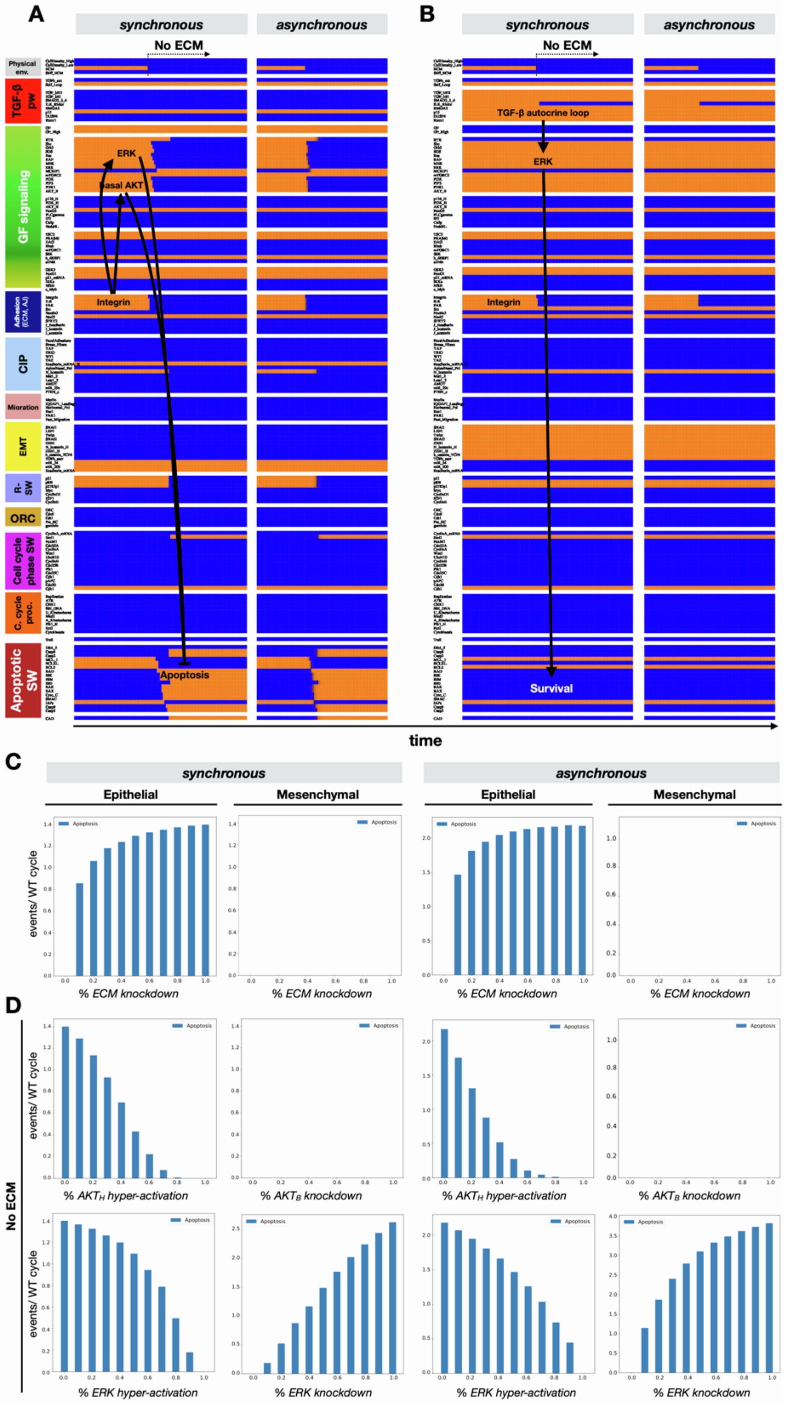

Figure S17. Loss of anchorage to ECM leads to apoptosis in epithelial cells but *TGFβ*-mediated survival in mesenchymal cells; related to Figure 5. A-B) Synchronous (*left*) and biased asynchronous (*right*) dynamics of regulatory molecule expression/activity in response to ECM detachment from soft ECM of (A) a growth-stimulated, isolated epithelial cell, and (B) a growth-factor starved, isolated mesenchymal cell. *X-axis:* time-steps; *y-axis:* nodes organized in regulatory modules; *orange/blue:* ON/OFF; *black/white labels & arrows:* molecular changes that drive MET. *Initial state for sampling runs:* isolated epithelial vs. mesenchymal cell in low mitogens on a soft ECM. **C)** Rate of apoptosis in isolated epithelial vs. mesenchymal cells on soft ECM in the presence of 95% saturating growth stimuli to an increasing loss of ECM adhesions. *Left/right:* synchronous/biased asynchronous update. **D)** *Left two panels:* rate of apoptosis in isolated epithelial cells upon complete loss of anchorage to an ECM together with increasing, forced activation of AKT_H (*top*), or *ERK* (*bottom*). *Right:* rate of apoptosis in isolated mesenchymal cells upon complete loss of anchorage to an ECM together with increasing, forced knockdown of AKT_B (*top*), or *ERK* (*bottom*) in the presence of 95% saturating growth stimuli (synchronous vs. biased asynchronous update). *Initial state for sampling runs:* isolated epithelial (*left*) or mesenchymal (*right*) cell in low mitogens on a soft ECM. *Total sampled live cell time:* 100,000 steps; synchronous update; *rate*: relative to normal cell cycle length.

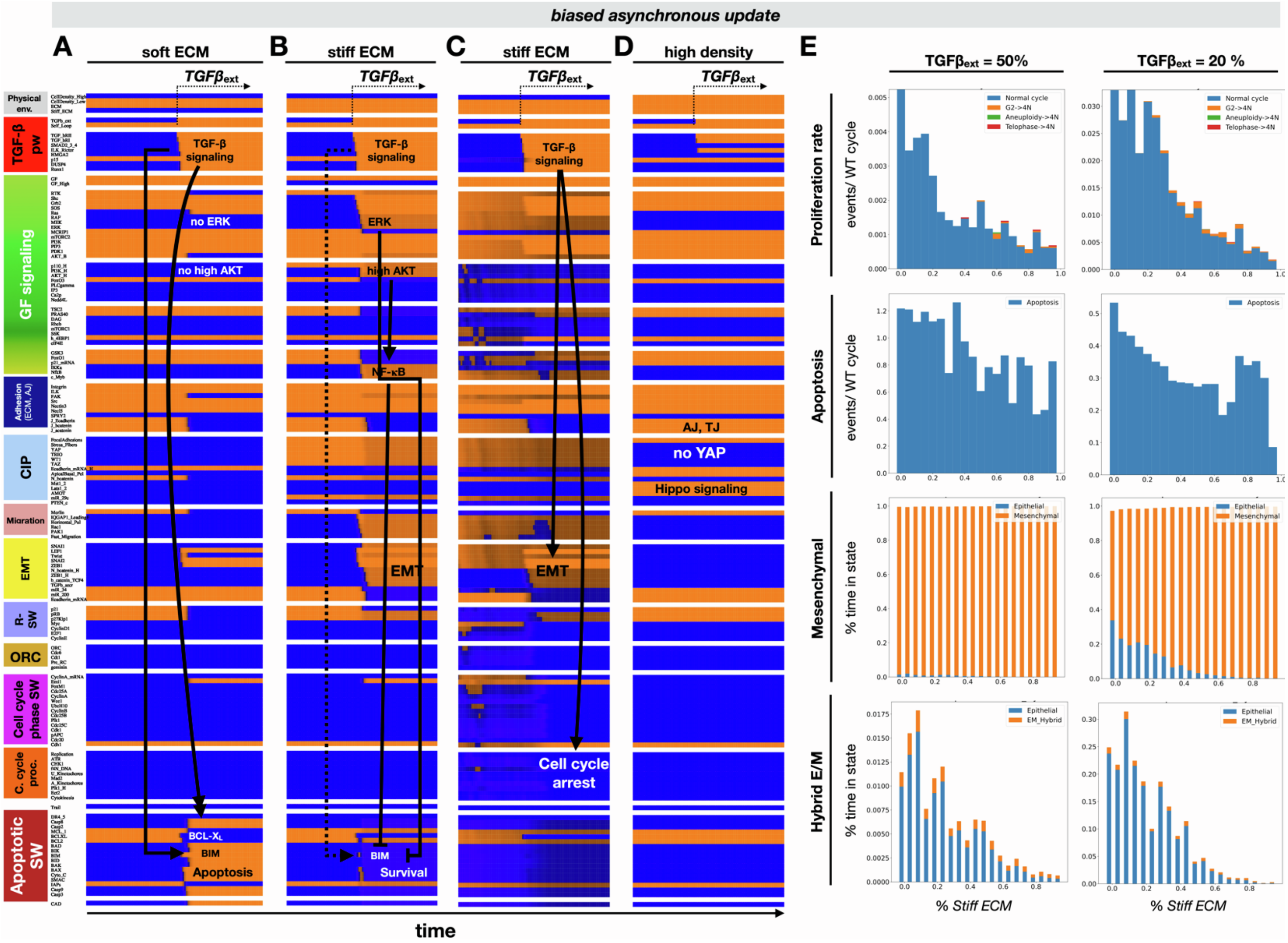

Figure S18. TGFβ-mediated apoptosis on soft ECM vs. EMT and cell cycle arrest on stiff ECM is robust to biased asynchronous update; related to Figure 6. A-D) Biased asynchronous dynamics of regulatory molecule expression/ activity during exposure to *TGFβ* of (A) growth-stimulated epithelial cells on soft ECM (medium density), (B) epithelial cells in low growth conditions on stiff ECM (medium density), (C) cycling hybrid E/M cells on stiff ECM (medium density), and (D) growth-stimulated but fully confluent (dense) epithelium on stiff ECM. *X-axis:* time-steps; *y-axis:* nodes organized in regulatory modules; *orange/black/blue color-scale:* average expression of each molecule across 1000 independent runs with biased asynchronous update (*orange* = all ON; *black* = 50% ON/OFF; *blue* = all OFF); *black/white labels & arrows:* molecular changes that drive apoptosis (A), EMT (B-C), or lack of response (D). **E)** Biased asynchronous response growth-stimulated epithelial cells at the edge of a monolayer to 50% (*left*) or 20% (*right*) external *TGFβ* at increasing ECM stiffness. *Top:* rate of normal cell cycle completion (*blue*) vs. G2 → G1 reset (*orange*), aberrant mitosis (*green*), or failed cytokinesis (*red*) followed by genome duplication, relative to the minimum cell cycle length (21 time-steps), shown as stacked bar charts. *Second from top:* rate of apoptosis (per minimum cell cycle length). *Third from top:* fraction of time spent in a mesenchymal (*orange*) vs. epithelial (*blue*) state. *Bottom:* fraction of time spent in a hybrid E/M (*orange*) vs. epithelial (*blue*) state. *Total sampled live cell time:* 100,000 steps; synchronous update. *Initial state for sampling runs:* dividing hybrid E/M at saturating mitogen exposure, at the edge of a monolayer on stiff ECM.

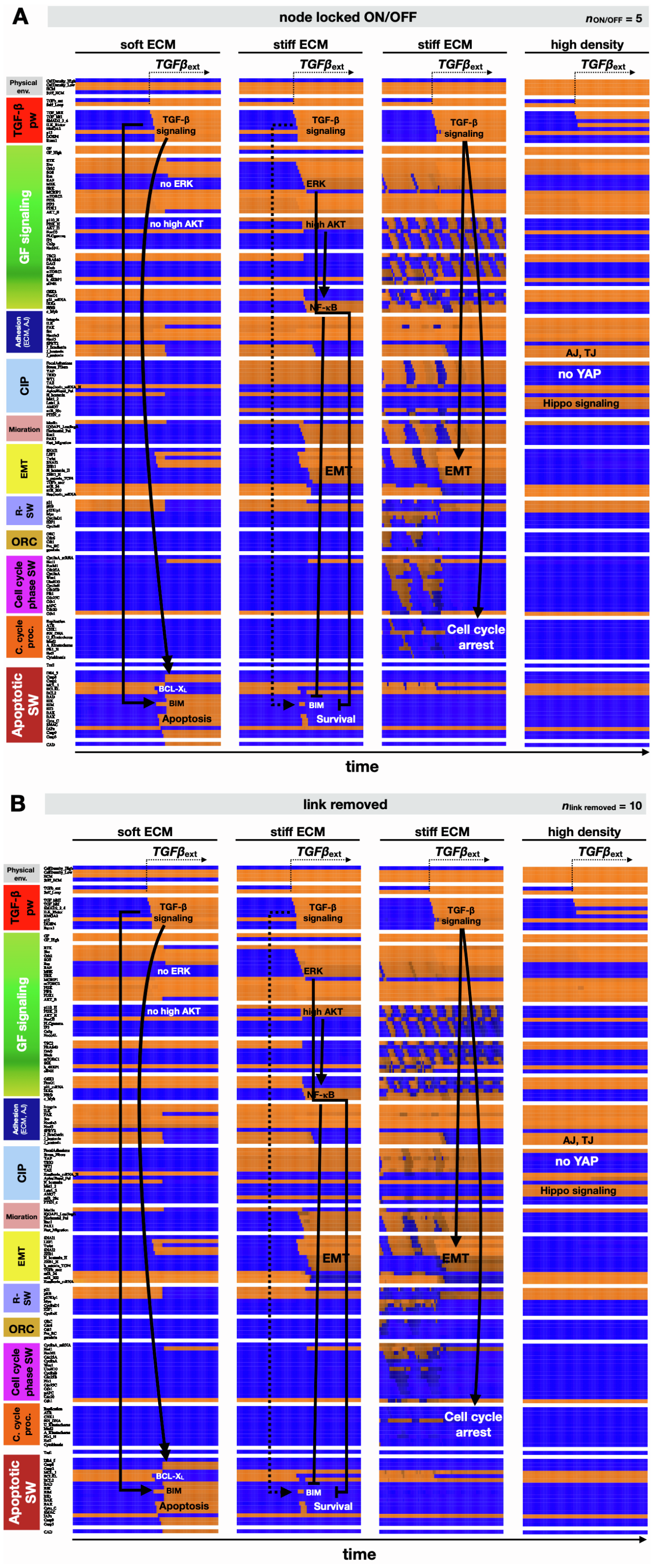

Figure S19. The model's mechanosensitive *TGFβ* response is robust to random mutations / errors in model construction; related to Figure 6. A-B) Average synchronous dynamics of regulatory molecule expression/activity during reversible exposure of an isolated, growth-stimulated epithelial cell to stiff ECM in an ensemble of mutant networks with (A) random nodes locked ON/OFF or (B) 10 random links removed, showing apoptosis on soft ECM (*1st panel*), EMT and cell cycle arrest on stiff ECM (*2nd and 3rd panels*), and no response at high cell density (*4th panel*). *X-axis*: time-steps; *y-axis:* nodes organized in regulatory modules; *orange/black/blue color-scale:* average expression of each molecule across 1000 time-courses from independently generated mutant models (*orange* = all ON; *black* = 50% ON/OFF; *blue* = all OFF; synchronous update); *black/white labels*: relevant molecular patterns.

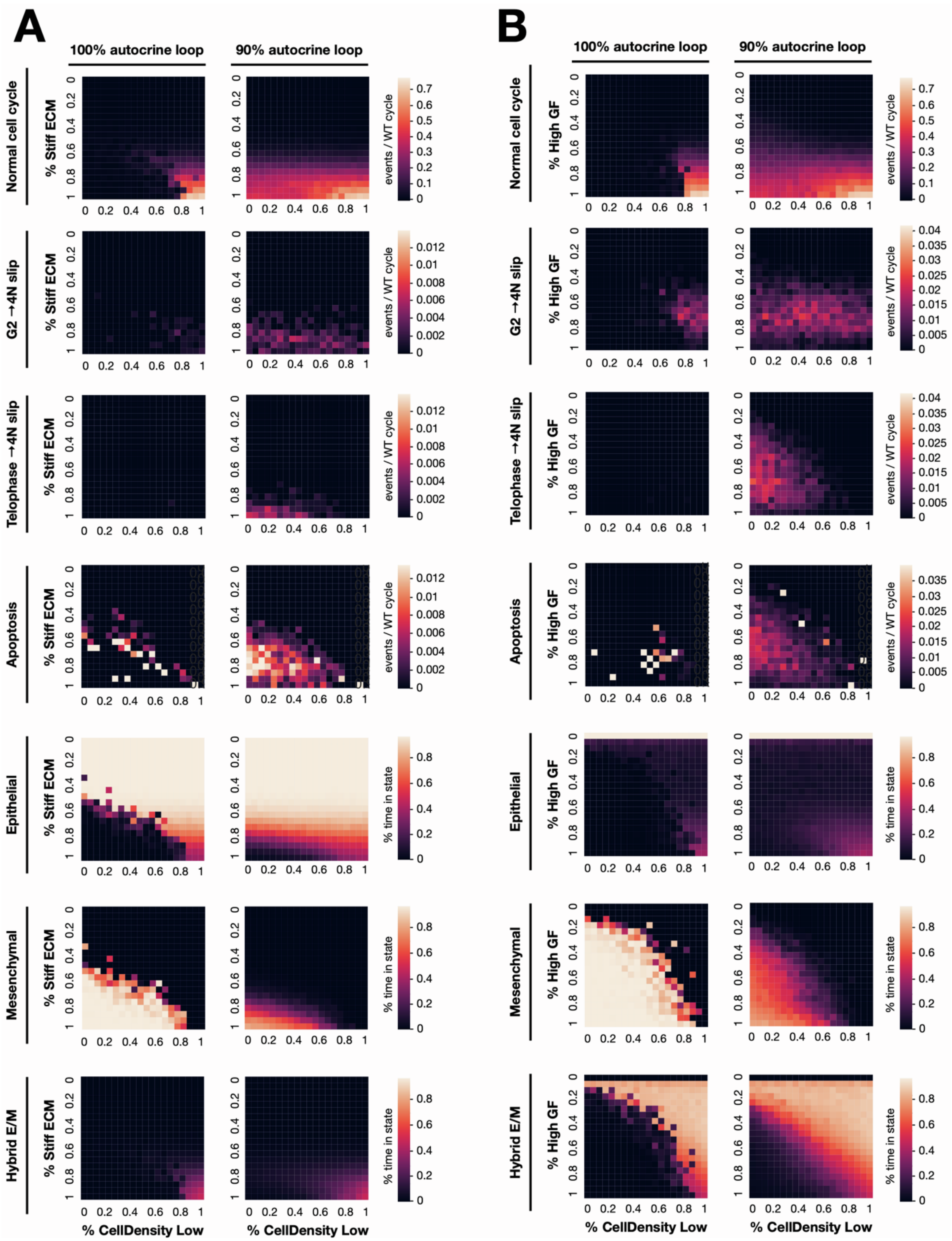

Figure S20. Below-saturating autocrine *TGFβ* signaling destabilizes the mesenchymal state and promotes proliferation; related to Figure 6. A) Behavior of 95% growth-stimulated epithelial cells at varying levels of density (*x* axis, from isolated to monolayer edge) and ECM stiffness (*y* axis) with 100% (*left*) or 90% (*right*) saturating autocrine *TGFβ* signaling capability (synchronous update). **B)** Behavior of epithelial cells on 95% stiff ECM at varying levels of density (*x* axis, from isolated to monolayer edge) and growth factor stimulus (*y* axis) with 100% (*left*) or 90% (*right*) saturating autocrine *TGFβ* signaling capability. *Top 4 panels:* normal cell cycle completion, G2 → G1 reset, failed cytokinesis followed by genome duplication, apoptosis (relative to minimum cell cycle length of 21 time-steps); *bottom 3 panels:* fraction of time spent in the epithelial, mesenchymal, hybrid E/M state. *Total sampled live cell time:* 100,000 steps; synchronous update; *initial state for sampling runs:* isolated epithelial cell in low mitogens on a soft ECM.
