# Supplementary material for: Boolean modeling of mechanosensitive epithelial to mesenchymal transition and its reversal
Source: iScience. 2023 Mar 2;26(4):106321. doi: 10.1016/j.isci.2023.106321 (PMC10030917; doi:10.1016/j.isci.2023.106321)

56 attractors for all Trail = 0 environments
(an additional 26 apoptotic attractors exist in Trail = 1 environments)

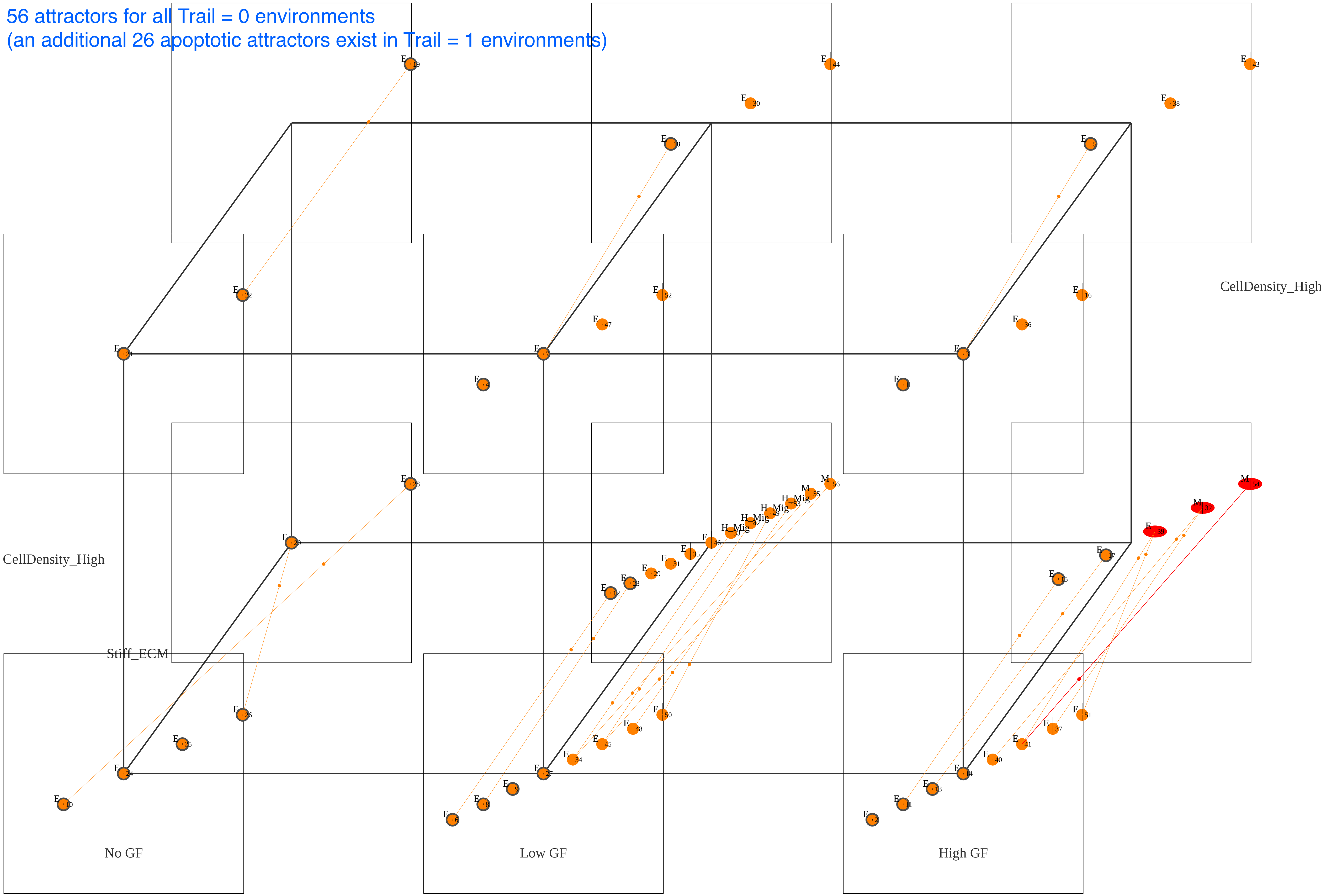

Supplement: Data S1. Detailed model description in human and machine-readable formats, related to STAR Methods — Included: File_S1_Model_justification.pdf (large table describing the biological evidence behind each node, link and logic gate of both models); Files S2-S3: EMT_Mechanosensing.dmms& EMT_Mechanosensing_TGFbeta.dmms (models in the .dmms format read bydynmod); Files S4-S5: File_S4_EMT_Switch.booleannet & File_S5_EMT_Mechanosensing.booleannet (models in BooleanNet format); Files S6-S7: File_S7_EMT_Mechanosensing.gml & File_S8_EMT_Mechanosensing_TGFbeta.gml (editable model network visualizations read by yED); File_S9_EMT_Mechanosensing_Steady_state_MAP.pdf (example output from ReganLabBooleanSims showing all model attractors organized by the cellular environment they are detected in); File_S10_VirtualExp_Phenotypes_for_STATS.txt (virtual experiment file example for ReganLabBooleanSims). [file mmc5.zip › File_S9_EMT_Mechanosensing_Steady_state_MAP.pdf]
